# The first 100 patients in the SUN(^_^)D trial (strategic use of new generation antidepressants for depression): examination of feasibility and adherence during the pilot phase

**DOI:** 10.1186/1745-6215-13-80

**Published:** 2012-06-08

**Authors:** Shinji Shimodera, Tadashi Kato, Hirotoshi Sato, Kazuhira Miki, Yoshihiro Shinagawa, Masaki Kondo, Hirokazu Fujita, Ippei Morokuma, Yoshio Ikeda, Tatsuo Akechi, Norio Watanabe, Mitsuhiko Yamada, Masatoshi Inagaki, Naohiro Yonemoto, Toshi A Furukawa

**Affiliations:** 1 Department of Neuropsychiatry, Kochi Medical School, Kohasu, Okoh-cho, Nankokushi, Kochi, 783-8505, Japan; 2 Aratama Kokorono Clinic, Suyama-cho, Mizuho-ku, Nagoya, 467-0066, Japan; 3 Harimayabashi Clinic, Harimaya-cho, Kochi, 780-0822, Japan; 4 Miki Clinic, 1-1-3 Hiranuma, Nishi-ku, Yokohama, 220-0023, Japan; 5 Shiki Clinic, Hongo, Meitou-ku, Nagoya, 465-0024, Japan; 6 Department of Psychiatry and Cognitive-Behavioral Medicine, Nagoya City University Graduate School of Medical Sciences, Mizuho-cho, Mizuho-ku, Nagoya, 467-8601, Japan; 7 Narumi Himawari Clinic, Narumi-cho, Midori-ku, Nagoya, 458-0801, Japan; 8 Department of Neuropsychopharmacology, National Institute of Mental Health, National Center of Neurology and Psychiatry, 4-1-1 Ogawa-Higashi, Kodaira, Tokyo, 187-8553, Japan; 9 Department of Epidemiology and Biostatistics, Translational Medical Center, National Center of Mental Health, National Centre of Neurology and Psychiatry, 4-1-1 Ogawa-Higashi, Kodaira, Tokyo, 187-8502, Japan; 10 Department of Health Promotion and Human Behavior, Kyoto University Graduate School of Medicine/School of Public Health, Yoshida Konoe-cho, Sakyo-ku, Kyoto, 606-8501, Japan

## Abstract

**Background:**

Initial glitches and unexpected inconsistencies are unavoidable in the early stage of a large, multi-centre trial. Adaptive modifications of the trial’s protocol and operational procedures to ensure its smooth running are therefore imperative. We started a large pragmatic, multi-centre, assessor-blinded, 25-week trial to investigate the optimal first- and second-line treatments for untreated episodes of nonpsychotic major depression in 2010 [Strategic Use of New generation antidepressants for Depression, abbreviated SUN(^_^)D] and would like to herein report an examination of the trial’s feasibility and adherence among the first 100 participants.

**Methods:**

We examined the participants’ characteristics, the treatments that were allocated and received during each step of the trial, and the quality of the outcome assessments among the first 100 patients enrolled in the SUN(^_^)D trial.

**Results:**

Of the 2,743 first-visit patients who visited the two collaborating centres between December 2010 and July 2011, 382 were judged as potentially eligible, and 100 of these patients provided written informed consent. These patients represented the whole spectrum of mild to very severe depression. Of the 93 patients who had reached Week 3 of the study by the end of July 2011, one withdrew consent for both the treatment and the assessment, and eight withdrew consent for the treatment only. Altogether, the primary outcomes were successfully assessed in 90 (96.8%) of the patients at Week 3. Of the 72 patients who had reached Week 9, three withdrew consent for the treatment, but 70 were successfully interviewed (97.2%). Of the 32 patients who had reached Week 25, 29 (90.5%) were successfully followed up. The inter-rater reliability of the assessments of the primary outcomes was nearly perfect and their successful blinding was confirmed. Minor modifications and clarifications to the protocol were deemed necessary.

**Discussion:**

Given the satisfactory feasibility and adherence to the study protocol and the minor modifications that were necessary, we conclude that the data obtained from the first 100 patients can be safely included in the main study. We now intend to accelerate the study by recruiting more collaborating centres and clinics/hospitals.

**Trial registration:**

ClinicalTrials.gov identifier: NCT01109693

## Background

A randomised clinical trial can only be started after its protocol and operational procedures have been fixed and written down in detail. Like any industrial product, however, some malfunctioning glitches and unexpected inconsistencies are unavoidable, especially during the early stages. Adaptively modifying the protocol and operational procedures to ensure the study’s smooth running is therefore imperative.

No guidelines exist, however, on how to implement this crucial step in the conduct of a clinical trial. One often-used method is to run a pilot study, separately from and before the formal study [1,2]. A drawback to this approach is that the data from this sample cannot in principle be merged into the full data set, especially if only limited aspects of the whole protocol are to be implemented and evaluated in the pilot study or if the randomisation is broken. Moreover, limiting the scope of the pilot study to avoid wasting valuable patient resources may ironically mean that all the study procedures cannot be fully tested in the pilot phase and that the same problem of initial adjustments may have to be worked through de novo only after the full-scale formal study has been initiated.

The Strategic Use of New generation antidepressants for Depression, or SUN(^_^)D for short, is a large pragmatic multi-centre, assessor-blinded, parallel-group trial to examine the optimal first- and second-line treatments of heretofore untreated episodes of nonpsychotic unipolar major depression [3]. It first compares the rapid titration strategy up to the maximum tolerable dosage versus the titration up to the minimum effective dosage of a selective serotonin-reuptake inhibitor (SSRI) antidepressant in the first-line treatment of depression in a cluster-randomised design. When the first-line treatment fails to achieve remission, it then attempts to compare three second-line treatment strategies, namely to augment the SSRI with a noradrenergic and specific serotonergic antidepressant (NaSSA), to switch from SSRI to NaSSA, or to continue several more weeks with SSRI, in an individually randomised comparison. The study will enrol 2,000 adult patients with untreated major depressive episodes seeking treatment at psychiatric clinics and hospitals at a number of regional centres from all over Japan.

In this large pragmatic psychiatric trial, we opted to include the feasibility examination in the main study itself. Namely, we decided to implement the whole study procedure from the very beginning, but at a limited number of regional centres, to examine the feasibility of the original protocol as well as the adherence of both the patients and the doctors to the protocol, and to modify, if necessary, the protocol and operational procedures as the first patients are enrolled in the study [3]. The current report describes the results of the feasibility and adherence examination among our first 100 patients, and details the required amendments to the SUN(^_^)D study protocol.

## Methods

### Ethics

The original study protocol was approved by the Institutional Review Board (IRB) at Nagoya City University Hospital in April 2010 by the Ethics Committee at Kyoto University Graduate School of Medicine in August 2010 and by the Ethics Committee at Kochi University Medical School in October 2010. The pilot phase of the SUN(^_^)D started its recruitment at two regional centres in Nagoya and Kochi on 6 December 2010, with the centre in Kyoto serving as the central office. Eligible patients provided written informed consent after receiving a full disclosure and explanation of the purpose and procedures of the study.

This trial has been registered at ClinicalTrials.gov as NCT01109693.

### Procedures

The full details of the procedures of the study are given in the published protocol [3] and we briefly present the overall flow of the trial, the participants’ eligibility criteria and the outcome measures here.

**Figure 1:**
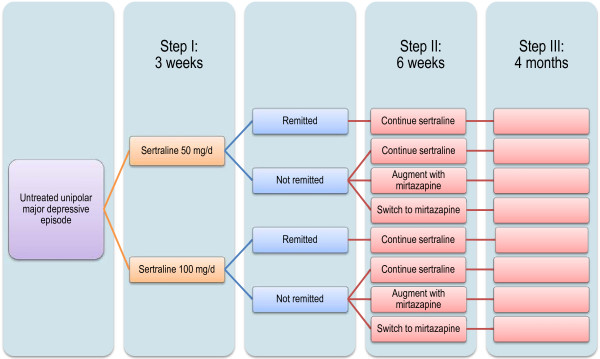
Flow diagram of the trial.

Figure 1 presents the overall procedure of the trial. The participants’ eligibility criteria are listed in Table 1. We chose sertraline as the first-line SSRI treatment because it was found to offer the best balance between efficacy and tolerability among the currently marketed anti-depressant drugs in Japan according to a recent multiple-treatments meta-analysis of 12 new-generation antidepressants [4]. The optimum titration strategy, however, has never been systematically examined in the literature. In step I, therefore, eligible and consenting patients will either receive sertraline titrated up to 50 mg/day or up to 100 mg/day unless prohibited by adverse effects for 3 weeks. We employed the cluster randomisation design for step I, i.e. the participating sites were randomised either to 50 mg/or 100 mg/d arms.

**Table 1:** Eligibility criteria

**Inclusion criteria**	**Exclusion criteria**
(1) The participant fulfils criteria for non-psychotic unipolar major depressive episode (DSM-IV) within 1 month before starting sertraline	1) Having taken antidepressants, mood stabilizers (lithium, valproate, carbamazepine, lamotrigine), antipsychotics, psychostimulants (methylphenidate, pemoline, atomoxetine), electroconvulsive therapy, rTMS, light therapy, or depression-specific psychotherapies (cognitive-behaviour therapy, interpersonal therapy) within 1 month before starting sertraline
(2) Age between 25 and 75 on the day when sertraline is started	(2) History of schizophrenia, schizoaffective disorder or bipolar disorder (DSM-IV) as judged by treating physician
(3) The major depressive episode is the focus of the treatment and the treating physician has judged sertraline to be its appropriate first-line drug	(3) Current dementia, borderline personality disorder, eating disorder or substance dependence (DSM-IV) as judged by treating physician
(4) Tolerability to sertaline has been ascertained after 3–16 days of treatment with sertraline 25 mg/day	(4) Physical diseases that may contraindicate treatment with sertraline or mirtazapine
(5) The participant is able to understand and sign written informed consent	(5) Allergy to sertraline or mirtazapine
(6) The participant is available on the phone for assessment of symptoms and side effects	(6) Terminal physical diseases
	(7) Women who are pregnant or breastfeeding (if there is a possibility of getting pregnant within 6 months of trial entry, participation is allowed only after providing signed consent to avoid pregnancy during the trial period)
	(8) Imminent high risk of suicide as judged by treating physician
	(9) Needing non-voluntary hospitalisation
	(10) High probability of changing hospital due to relocation, etc., within 6 months of trial entry
	(11) Cohabiting family members of research staff members of the trial
	(12) Inability to understand written Japanese

When sertraline fails as a first-line treatment to achieve remission, one natural choice is to switch to the antidepressant found to be the most efficacious, albeit with reduced acceptability, in the same meta-analysis, namely mirtazapine [4]. Because this NaSSA has a different neurochemical profile than SSRI and has been found to work in synergy when combined with SSRI in a number of trials [5-7], another option is to augment SSRI with mirtazapine. These two second-line options are to be compared with continuing with sertraline for 6 more weeks in step II. If the patients remit after receiving the first-line treatment, they are to continue with the first-line treatment in step II.

Step III is a 16-week extension of steps I and II, in which the doctors and the patients are free to continue their step II treatments or to choose whatever treatments they deem appropriate. This stage represents the important continuation phase in the treatment of major depression, because almost every patient is advised to receive this continuation treatment [8]. In step III we therefore attempt to examine which of the first- and second-line treatments will be continued to sustain remission.

The primary outcome measures include the Patient Health Questionnaire-9 (PHQ-9) [9] and the Frequency, Intensity and Burden of Side Effects Rating (FIBSER), as assessed by the central raters at weeks 1, 3, 9 and 25. The assessment is conducted via telephone while the assessors are blinded to the treatment status of the patients. The inter- and intra-rater reliability of the primary outcome measures was examined by having all six raters re-assess the audio recordings of the 20 telephone interviews. The secondary outcomes included the Beck Depression Inventory-II [10], rated by the patients themselves.

As we had declared in the protocol [3], the analyses of the pilot phase were to be performed without knowledge of the allocated treatments and would not involve any statistical comparison between randomised arms. Unless a major change in the study protocol was required, the participants in the pilot phase would therefore be eligible for inclusion in the main study.

### Analyses

In this study the focus was placed on how feasible the original protocol was and how adherent the doctors and the patients were to steps I through III of the study. All the data had been provided to the researchers from the data centre without any information linking the treatment allocations and assessment results. We first reported the demographic and clinical characteristics of the screened patients as well as the finally enrolled patients to examine the efficiency of our recruitment procedure as well as to ascertain whether the intended types of patients had been recruited into the study. We then examined whether the randomisation had been successful, if the treatments as stipulated in the protocol had been adhered to and if the assessments had been made with satisfactory follow-up rates in each step of the trial.

The quality of the assessments is of paramount importance in a trial. We therefore examined the reliability of our primary outcomes and whether the blinding had been successfully implemented in the study.

Finally, we also reported the important safety aspects among our first 100 patients.

## Results

### Participating sites

Two regional centres participated in the pilot phase of the SUN(^_^)D. One regional centre consisted of one general hospital and five private psychiatric clinics, while the other centre included four hospitals and two private psychiatric clinics.

### Participants

**Figure 2:**
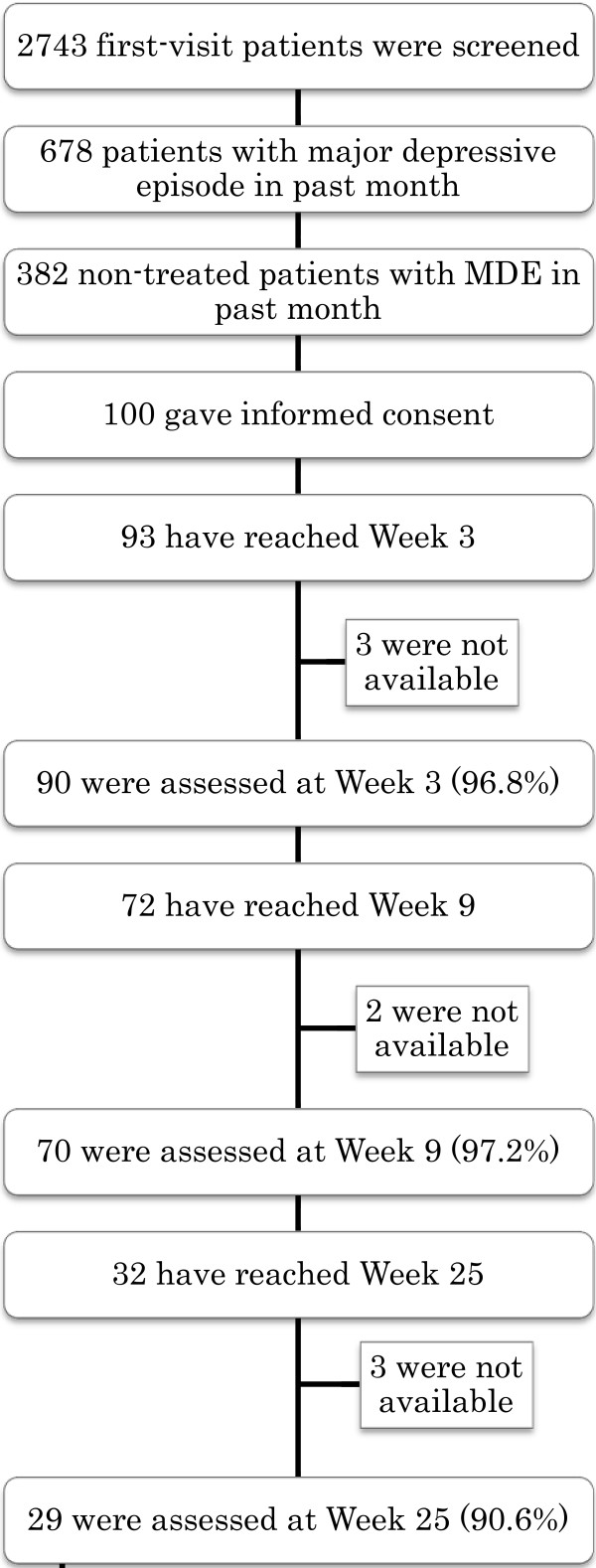
Flowchart of the participants as of the end of July 2011.

Altogether 2,743 first-visit patients visited the participating sites between 6 December 2010 and 31 July 2011. After undergoing standard psychiatric consultation and formal diagnosis with the help of the semi-structured interview PRIME-MD [9], 382 patients were judged to have suffered from a major depressive episode during the past month and to have had no treatment. One hundred of these patients were subsequently judged to meet all the eligibility criteria, provided written informed consent after full explanation of the purpose and procedures of the study, and were enrolled in the study (Figure 2).

**Figure 3:**
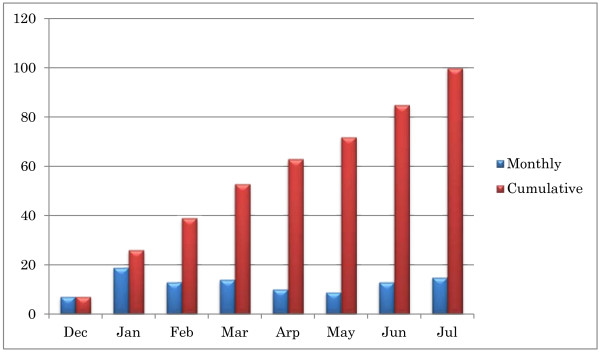
Monthly recruitment of the participants.

**Figure 4:**
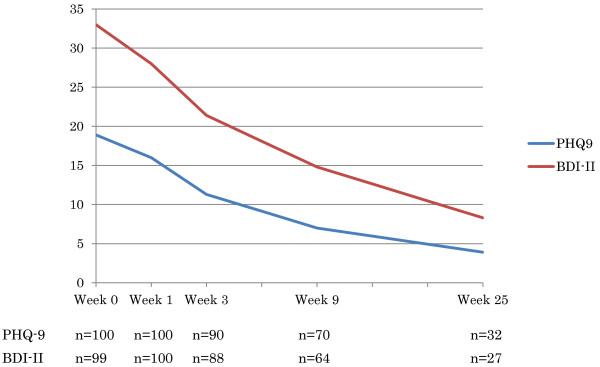
Average course of depression severity of the entire cohort.

Figure 3 depicts the monthly recruitment of the participants for the first 8 months of the study. On average, 12.5 patients were enrolled per month (Figure 4).

Table 2 describes the basic demographic characteristics of these participants. The subjects were approximately equally divided into both sexes and represented the whole age range as specified in the protocol, with a peak age occurring in the 5th decade. Slightly more than half of the patients had received education at a college or university level or beyond. About half the subjects were employed at the time of their entry into the study, but about a third were on sick leave.

**Table 2:** Demographic characteristics of the first 100 patients in SUN(^_^)D

**Age, years**	**42.4 (SD = 12.7, range: 25–75)**
Sex	56 women/44 men
Education	8 with junior high school or equivalent
	30 with senior high school or equivalent
	53 with junior college or university level
	9 with above university level
Job	36 employed full-time
	8 employed part-time
	28 on sick leave
	16 housewives
	2 students
	10 with no employment
Marriage	34 single, never married
	13 single, divorced or separated
	2 single, deceased
	51 married

Table 3 gives the clinical characteristics of the participants. The average age of the participants at the onset of the depressive disorder was in their 30s, some 5 years earlier than their age at first visit. For half of the cohort, the index episode was their first major depressive episode, but the others had had up to four previous episodes. The median length of the episode before the hospital visit was 2.5 months, with a range of between 0.5 to 120 months. The participants’ average depression scores were 18.9 for the PHQ-9 and 33.0 for the BDI-II, but the score varied between 8 to 27 and 14 to 52, respectively, thus representing the whole spectrum of mild to very severe depression [11].

**Table 3:** Clinical characteristics of the first 100 patients in SUN(^_^)D

**Age of onset at first episode, years**	**37.3 (SD = 14.0, range: 10 to 75)**
No. of previous depressive episodes	50 with no episode
	32 with one episode
	10 with two episodes
	2 with three episodes
	6 with four episodes
Length of current episode, months	6.0 (SD = 13.6, median = 2.5, range: 0.5 to 120)
Out- or inpatient status at time of entry into the study	100 outpatients
PHQ-9	18.9 (SD = 3.7, range: 8 to 27) at baseline
	16.0 (SD = 4.5, range: 7 to 25) at week 1
BDI-II	33.0 (SD = 8.4, range: 14 to 52) at baseline
	28.1 (SD = 9.8, range: 6 to 54) at week 1
Physical conditions	66 with no physical comorbidity
	6 with hypertension
	5 with diabetes mellitus
	4 with hypercholesterolemia
	2 each with hyperthyroidism, glaucoma, goitre, headache
	(including migraine) or arthritis
	1 each with hepatitis C, Meniere’s disease, asthma, haemorrhoids, kidney stone, esophagitis, arrhythmia, or collagen disease

### Adherence to the protocol in step I

#### Treatment allocation at the beginning of step I

Of the 100 initial participants, 16 were recruited at the five sites allocated to the sertraline 50 mg/day arm and the remaining 84 were recruited at seven sites allocated to the sertraline 100 mg/day arm. This imbalance in the number of recruited patients was due to one site that recruited the largest number of patients.

#### Treatment received by the end of step I

Of the 100 patients who had provided their written informed consent at week 1 by the end of July (the observation period for this feasibility study), 93 would have reached week 3 during this observation period. We then examined the treatments that these patients received in step I.

Of these 93 patients, 16 were in the sertraline 50 mg/day arm and 77 were in the sertraline 100 mg/day arm. None of the 16 patients allocated to the sertraline 50 mg/day arm withdrew their consent and all these patients had reached the designated dosage by week 3.

Of the 77 participants allocated to the 100 mg/day arm, one withdrew consent to both the protocol treatment and to undergoing further assessments, and was therefore dropped from the study. Eight additional patients withdrew consent to the protocol treatment by week 3, but consented to further assessments at weeks 3, 9 and 25: one patient had improved considerably and decided to stop the medication, one patient decided to stop the drug because of side effects, one patient was hospitalised because of depressive stupor, one patient started working and could not come to the clinic at the appointed times, two patients moved, and two patients withdrew consent for unknown reasons. Of the 68 patients allocated to the sertraline 100 mg/day arm and whose consent to the treatment remained valid, 10 did not reach the intended dosage by week 3 and were only receiving 75 mg/day.

None of the participants received the prohibited concomitant treatments during step I.

#### Assessment at the end of step I

Regardless of the treatments received, 93 patients should have received their week 3 assessments during the present study’s observation period. Unfortunately, two of the eight patients who withdrew their consent to the protocol treatment but still agreed to further assessments were unreachable at week 3. Thus, altogether three patients (one who withdrew consent to treatment and to further assessment, and two who withdrew consent to treatment but not to assessment) did not undergo PHQ-9 and FIBSER assessments at week 3. The follow-up rate was 96.8% (90/93).

### Adherence to the protocol in step II

#### Treatment allocation at the beginning of step II

Of the 93 patients who reached week 3 by end of July, 9 had withdrawn consent to treatment and 84 patients could therefore be randomised at week 3. Seven of these patients scored 4 or less on the PHQ-9 at week 3 and were therefore prescribed the same antidepressant in step II as in Step I. The nine patients who had withdrawn their consent to treatment received treatments of their and their doctors’ choice.

Seventy-seven patients were then randomised in a 1:1:1 manner to continue sertraline, to receive mirtazapine augmentation, or to switch from sertraline to mirtazapine. The allocated numbers of patients were 26, 24 and 27, respectively, and the randomisation procedure seemed to be result in equally numbered and well balanced groups for the two stratification variables.

#### Treatment received by the end of step II

Of the 77 patients randomised at week 3, 62 would have reached week 9 by the end of July (the observation period for this feasibility study).

Twenty-two had been allocated to continue sertraline and should have reached week 9; only one of them dropped out of the treatment prematurely. Nineteen had been allocated to receive mirtazapine augmentation of sertraline and should have reached week 9; all of them received the protocol treatment (i.e. 50–100 mg/day of sertraline plus 7.5 to 45 mg/day of mirtazapine). Twenty-one patients had been allocated to the switching-to-mirtazapine group; two of them stopped the treatment prematurely but all the others had been successfully switched to mirtazapine 15–45 mg/day. None of the patients received the prohibited concomitant treatments during step II.

#### Assessment at the end of step II

Of the original 100 patients recruited into the study, 72 should have reached their week 9 assessments by the end of July. In step II three patients dropped out from the protocol treatment but none of them withdrew their consent to further assessment (the one participant who withdrew consent to both the treatment and the assessments in step I had not reached his week 9 date by end of July). However, one patient who had been hospitalised in step I and another who had been allocated to the continuation of sertraline group in step II but stopped treatment prematurely despite medical advice was unreachable at week 9. The follow-up rate was therefore 70/72, or 97.2%.

### Adherence to the protocol in step III

#### Treatments received in step III

This feasibility and adherence study did not closely examine the contents of the treatments provided to the patients in step III because, according to our protocol, there were no prohibited co-interventions for this period, and the adherence to protocol treatments presented no concern in this step. The contents of the treatments provided were recorded in the data set; at end of week 25, 53% of the patients reported that they were continuing to receive the protocol treatment assigned to them in step II.

#### Assessment at the end of step III

Of the original 100 patients recruited into the study, 32 patients should have had their week 25 assessments by the end of July. So far we have been able to follow-up 29 of these patients (90.6%).

### Primary outcomes

#### Inter- and intra-rater reliability of the primary outcomes

Table 4 shows the ANOVA intra-class correlation coefficients for the individual items of the PHQ-9 and the FIBSER, and for the PHQ-9 total scores, based on the re-assessments made by six interviewers of ten audio recordings of telephone interviews. The inter-rater reliability among the six raters was perfect to nearly perfect.

**Table 4:** Inter-rater reliability for PHQ-9 and FIBSER

**PHQ-9**	**Item 1 (anhedonia)**	**0.953 (0.915 to 0.978)**
	Item 2 (depressed mood)	1.000 (−)
	Item 3 (sleep problem)	1.000 (−)
	Item 4 (anergia)	1.000 (−)
	Item 5 (appetite problem)	0.992 (0.985 to 0.996)
	Item 6 (guilt feelings)	1.000 (−)
	Item 7 (concentration problem)	0.984 (0.970 to 0.993)
	Item 8 (psychomotor symptoms)	1.000 (−)
	Item 9 (suicidal wishes)	1.000 (−)
	Total score	0.998 (0.996 to 0.999)
FIBSER	Item 1 (adherence)	1.000 (−)
	Item 2 (frequency of side effects)	1.000 (−)
	Item 3 (strength of side effects)	1.000 (−)
	Item 4 (burden of side effects)	0.996 (0.993 to 0.998)

The intra-rater (test-retest) reliability of the telephone assessments, that is the agreement between the interviewer’s ratings during the telephone interview and those made by the same interviewer when he/she listened to his/her own recordings 1 to 2 weeks later, was perfect for all the items.

#### Assessors’ blindness to allocated treatments

The assessors’ blindness to the allocated treatment was evaluated by tabulating their guessed treatments and the actually allocated treatments.

Table 5 presents such agreement for step I allocations and Table 6 presents the same data for the step II allocations. Because the assessors were blinded to the timing of the assessments, they sometimes made mixed guesses for both step I and step II. The agreement for the correct versus incorrect guesses for the step I treatments was a kappa of −0.19 (95% CI: -0.34 to −0.04) and that for the step II treatments was a kappa of −0.04 (−0.14 to 0.06).

**Table 5:** Examination of assessor blindness: Treatment guesses for week 3 assessments

**Guess**	**Actual**	
	50 mg/day	100 mg/day
50 mg/day at week 3	5	31
100 mg/day at week 3	8	23
Continue sertraline at week 9	2	15
Augment with mirtazapine at week 9	-	4
Switch to mirtazapine at week 9	-	1
Remitted at week 9	1	-

**Table 6:** Examination of assessor blindness: Treatment guesses for week 9 assessments

**Guess**	**Actual**			
	Continue sertraline	Augment with mirtazapine	Switch to mirtazapine	Remitted
50 mg/day at week 3	1	2	3	2
100 mg/day at week 3	9	6	5	-
Continue sertraline at week 9	6	8	8	1
Augment with mirtazapine at week 9	4	2	4	-
Switch to mirtazapine at week 9	-	-	1	-
Remitted at week 9	1	1	-	1

### Overall outcomes

Figure 3 presents the overall outcomes of our cohort as a group, undivided for the treatments allocations.

The number of remitters (PHQ-9 = <4) were 10, 21 and 16 at week 3, 9 and 25, respectively. These figures correspond to 10.8% (10/93), 29.2% (21/72) and 50.0% (16/32) of the intention-to-treat sample, respectively.

### Adverse events and change in diagnoses

No serious adverse event has been reported among the first 100 patients in the first 8 months of the SUN(^_^)D trial.

Suicidality was assessed according to the Columbia Classification Algorithm of Suicide Assessment (C-CASA) [12] at week 9 and at week 25 retrospectively by the treating psychiatrists. No case of a completed suicide, suicide attempt or preparatory act toward imminent suicidal behaviour was reported. Two patients expressed strong suicidal ideation to the doctor and/or the families.

One patient presented with depressive stupor and was hospitalised during step I. One patient presented with a hypomanic episode during step II. No other changes in the diagnoses have been reported.

### Modifications to the original protocol and procedures

Through the initial pilot phase of SUN(^_^)D, only the following minor modifications to the protocol and the operational procedures were necessary.

1) We added repetitive transcranial magnetic stimulation (rTMS), light therapy and lamotrigine as prohibited treatments both before entry to the trial and in steps I and II. We had overlooked rTMS and light therapy in the original protocol. Lamotrigine newly appeared on the Japanese market after the study was begun.

2) We explicated the eligibility criteria by adding, “The major depressive episode is the focus of the treatment” because we encountered patients with major depressive episodes comorbid with anxiety disorder, for whom the latter would clinically be the target condition of treatment. We reasoned such cases should be excluded from our trial.

3) We allowed more flexible titration schedules for step I. In the original protocol there was only one anticipated titration schedule for the sertraline 100 mg/day arm, namely 50 mg/day at week 1 titrated up to 100 mg/day at week 2. In the revised protocol, any duration of any dosage was permitted at week 1, such as 75 mg/day for a week or 50 mg/day for 3 days plus 75 mg/day for 4 days, with the minimum requirement of reaching 100 mg/day at week 2.

4) We also accepted 7.5 mg/day of mirtazapine in step II. In the original protocol, mirtazapine was to be prescribed at a dosage of between 15 and 45 mg/day.

5) A number of new reporting forms were prepared and updated to gather the necessary information more systematically and more efficiently.

## Discussion

We started the first large-scale pragmatic clinical trial of antidepressant treatment in Japan in December 2011. This trial is an assessor-blinded, parallel-group, multi-centre trial that aims to enrol 2,000 patients with heretofore untreated unipolar major depressive episodes to determine the optimum first- and second-line antidepressant treatment strategies. The current study reports on the feasibility and adherence of the study protocol and operational procedures among the first 100 patients.

### Patient enrolment and characteristics

At the 12 psychiatric clinics and hospitals associated with two centres in Nagoya and Kochi, we screened approximately 2,700 first-visit patients, of whom approximately 400 were judged as possibly being eligible and 100 were finally entered in the study after providing written informed consent over the course of the initial 8 months of the study. We consider that this is a respectable figure and calculate that, if we can liaise with six more centres, each associated with five clinics or hospitals in the main study, we would be able to enrol eight centres * 6 patients/month * 36 months = approximately 1,800 patients in 3 years.

If we can continue with the one out of four recruitment rate and the sample characteristics as shown in Tables 2 and 3, we can be confident that our cohort will be representative of mildly to very severely depressed patients seeking initial treatment for their untreated depressive episodes. For example, the female:male ratio of 56:44 = 1.3, though much lower than the 2:1 ratio usually observed in American or European samples, is close to the ratio of 1.4 reported in a former Japanese study of a similar inception cohort of untreated major depression [13].

### Randomisation

The randomisation for step I was balanced at the level of clinics and hospitals between 50 mg/day and 100 mg/day arms, but was unbalanced at the individual level because of one very actively recruiting clinic. While this imbalance in the numbers of patients allocated to the two arms may slightly decrease the statistical power, it is unlikely to undermine the internal validity of step I. Moreover, we expect that the treatment allocation will eventually be balanced as we enrol more participating centres and clinics in the main study.

The randomisation for step II was well balanced among its three arms and its strata.

### Treatment adherence

In step I, of the 93 patients who should have reached the end of this step, nine (9.7%) withdrew their consent to the protocol treatment. Of the remaining 84 patients, 74 (88.1%) received the treatments as required by the protocol.

In step II, of the 62 patients who had been randomised to one of the three treatment arms and who should have reached the end of this step, only three (4.8%) withdrew consent to the protocol treatments. When the patients continued to receive treatment in any one of the three arms, no protocol deviation occurred.

A voluntary withdrawal rate from the assigned treatment of between 5-10% and a protocol deviation rate of between 0-10% if continued would be acceptable figures in a large pragmatic trial [14,15].

### Follow-up assessments

In the study protocol we sharply distinguished between withdrawal from the protocol treatments and that from the follow-up assessments, and invited the patients to cooperate with the follow-up assessments even if they had voluntarily chosen to withdraw from the assigned treatments. As a result, we have so far been able to successfully assess 96.8%, 97.2% and 90.6% of the intention-to-treat sample at week 3, 9 and 25, respectively. We accept that the follow-up rate at week 25 needs to be improved.

In this study we were able to establish the inter- and intra-rater (test-retest) reliability of our primary outcomes and also to ascertain the successful blinding of the assessments thus made. This trial therefore represents one of the fewer instances where blinding was appropriately tested and confirmed [16].

### Safety

We encountered no unexpected or concerning safety issues among our first 100 patients.

## Conclusion

Based on the present feasibility and adherence examinations of the pilot study phase of the SUN(^_^)D study, we conclude that the study protocol [3] can be implemented as originally envisaged with some minor modifications only and that the data from the first 100 patients can therefore be safely and validly included towards the main study. We will continue with the pilot phase of the study as we had originally set out in the protocol until the first 200 patients have completed their 25-week follow-ups, but we are now confident that we can speed up recruitment of collaborating centres and clinics/hospitals, thereby accelerating not only the pilot phase but also the entire study itself.

## Competing interests

SS has received speaking fees and/or research funds from Astellas, Dainippon-Sumitomo, GlaxoSmithKline, Janssen, Lilly, MSD, Otsuka, Pfizer, Shering-Plough, Shionogi and Yoshitomi. KM has received speaking fees from Astellas, Dainippon-Sumitomo, GlaxoSmithKline, Janssen, Lilly, Meiji, Otsuka, Pfizer and Shering-Plough. YS has received a speaking fee from Meiji. MK has received speaking fees from Lilly and Otsuka. HF received speaking fees from GSK, Novartis, Janssen, Lilly and Mochida. TA has received speaking fees and/or research funds from Astellas, Astra-Zeneca, Bristol-Meyers-Squib, Daiichi-Sankyo, Dainippon-Sumitomo, Eisai, GlaxoSmithKline, Janssen, Kyowa-Hakko-Kirin, Lilly, Meiji, Otsuka, Pfizer, Sanofi-Aventis, Shionogi, Yakult, MSD, Novartis Pharma and Chugai. NW has received speaking fees and/or research funds from Dainippon-Sumitomo, GlaxoSmithKline, Lilly, Otsuka, Pfizer, Asahi-Kasei and Shering-Plough. MI has received a speaking fee from Lilly. NY received royalties from Seiwa-Shoten. TAF has received honoraria for speaking at CME meetings sponsored by Asahi Kasei, Eli Lilly, GlaxoSmithKline, Kyorin, Meiji, Mochida, MSD, Otsuka, Pfizer, Shionogi and Tanabe-Mitsubishi. He is on advisory board for Pharmaceuticals and Medical Devices Agency, Sekisui Chemicals and Takeda Science Foundation. He has received royalties from Igaku-Shoin, Seiwa-Shoten and Nihon Bunka Kagaku-sha. All the other authors have no competing interests to declare.

## Authors’ contributions

TAF conceived the study. SS and TAF prepared the original manuscript. TK, HS, KM, YS and TI collaborated in data collection; these authors along with TA, NW, MY, MI and NY participated in the refinement of the manuscript. NY is the trial statistician. All authorsread and approved the final manuscript.
